# The role of mRNA degradation in immunity and inflammation

**DOI:** 10.1186/ar3562

**Published:** 2012-02-09

**Authors:** Shizuo Akira

**Affiliations:** 1 Laboratory of Host Defense, WPI Immunology Frontier Research Center, Osaka University, 3-1 Yamadaoka, Suita City, Osaka 565-0871, Japan

## 

The innate immune system is an evolutionally conserved host defense mechanism against pathogens. Innate immune responses are initiated by pattern recognition receptors (PRRs), which recognize specific structures of microorganisms. Among them, Toll-like receptors (TLRs) are capable of sensing organisms ranging from bacteria to fungi, protozoa and viruses, and play a major role in innate immunity. Individual TLRs recognize different microbial components, and give rise to different patterns in gene expression.

We are now focusing on the role of genes induced in response to TLR stimulation, particularly the genes that are rapidly induced in a MyD88-dependent manner within 30 min after LPS stimulation. Among them, we have recently identified a novel gene named Zc3h12a which has a CCCH-type zinc finger domain. The knockout mice developed spontaneous autoimmune diseases accompanied by splenomegaly and lymphadenopathy. Subsequent studies showed that Zc3h12a is a nuclease involved in destabilization of IL-6 and IL-12mRNA. We renamed it Regulatory RNase-1 (Regnase-1) based on the function.

We recently found that the IKK complex controls *Il6 *mRNA stability by phosphorylating Regnase-1 in response to IL-1R/TLR stimulation. Phosphorylated Regnase-1 underwent ubiquitination and degradation. Regnase-1 re-expressed in IL-1R/TLR-activated cells exhibited delayed kinetics, and Regnase-1 mRNA was found to be negatively regulated by Regnase-1 itself via a stem-loop region present in the Regnase-1 3' untranslated region. These data demonstrate that the IKK complex phosphorylates not only IkBalpha, activating transcription, but also Regnase-1, releasing the "brake" on *Il6 *mRNA expression.

